# Supplementary material for: The detection dogs test is more sensitive than real-time PCR in screening for SARS-CoV-2
Source: Commun Biol. 2021 Jun 3;4:686. doi: 10.1038/s42003-021-02232-9 (PMC8175360; doi:10.1038/s42003-021-02232-9)
Supplement: Supplementary file 2 — Supplementary Information [file 42003_2021_2232_MOESM2_ESM.pdf]

Supplementary Information

"The Detection Dogs Test is more sensitive than Real-Time PCR in screening for SARS-CoV-2"

By

Mohammed Hag-Ali, Abdel Salam Alshamsi, Linda Boeijen, Yasser Mahmmod, Rashid Manzoor, Harry Rutten, Marshal M. Mweu, Mohamed El-Tholoth, Abdullatif Alteraifi AlShamsi

This "Supplementary Information" file provides more details on the following sections of the manuscript;

1. Methods section, including all aspects of K9 dogs' selection and training protocols.
2. Results and discussion section regarding the Bayesian analysis studies.

Supplementary Methods Section

I- K9 dogs selection

Four male Federal Customs Explosive detection dogs (K9) were selected for the training to screen for SARS-CoV-2 using axillary sweat samples. One dog was a Belgian Malinois, two dogs were cross breeds (Belgian Malinois and German Shepherd) and one was German Shepherd. Dogs were fully trained and certified twice for explosive detection. The dog teams (dog and handler) have been working together for over two years. Dogs have been trained in search behaviour; indication of explosive scents in multiple training scenarios (scent boxes, scent wall, different types of buildings, areas, cargo, luggage and packages and vehicles), including operations at multiple Federal Customs locations. All dogs were medically and physically checked by a veterinarian before the start of training. We selected experienced explosive detection dogs (K9) and their handlers because the dogs already have an excellent search pattern and they understand how to indicate the scent of a variety of explosives. At the start of the trial, the dogs only needed to imprint and memorize the additional scent of sweat samples from SARS-CoV-2 positive patients. We selected male dogs to avoid issues with coming into oestrous cycle during the trial because the qualified females in the current group of dogs were not spayed. Narcotics detection dogs were excluded in our study to avoid the false positive results in

individuals receiving prohibited narcotics even though we were not aware of any scientific research to prove this; hence, we decided to avoid this risk.

The selected dogs showed the following desirable traits: 1. High search drive. 2. High focussing ability. 3. Obedient to handler and trainer commands. 4. Sociability to work in public environments. 5. Not aggressive without reasons or nervous to environmental changes. 6. Independence (i.e. have the ability to search alone). 7. Friendly to people. 8. A very good ability to pinpoint targeted odour. 9. Methodical and detailed search behaviours. 10. Playfulness. 11. Easy adaptation to a new environment.

II- Biosafety Considerations

Centers for Disease Control and Prevention (CDC) - stated that dogs are not playing an efficient role in SARS-CoV-2 spreading. In contrast to the previous work by Jendrny et al.[2] that identified SARS-CoV-2 in saliva and tracheobronchial secretions using trained dogs, our study used sweat that is not a route for SARS-CoV-2 excretion from infected patients[3], rendering the sample to be non- infectious[4]. In January 2021, PCR tests were conducted on fifty Federal Customs working dogs including the dogs of this study; all results came back negative.

III- Sampling Protocol

Licenced and trained healthcare professionals collected the sweat samples for training purpose from volunteers that tested positive or negative by PCR. The technical staff were educated to work using personal protective equipment (PPE) and not to contaminate the samples with their own odours. The samples that were collected in the hospital were transported the same day to the Federal Customs K9 facility. The positive and negative samples were transported separately in medical coolers with ice packs, but under the same conditions. After arriving in the Federal Customs K9 facility, the samples were stored in the fridge and used the same day. All samples were taken out of the fridge to come to 'room temperature' before presenting to the dogs.

ii. The operational samples were collected by the participant, under supervision of an assigned nurse or medical doctor explaining the procedure and making sure that the sampling protocol was followed. The samples were stored in airtight containers (see Figure 3.) with the data of each participant, kept in a holding area next to the testing room, and were presented to dogs within a maximum of two hours after collection.

IV- Training Protocol

A. Instructor, trainer and handler selection:

1. **Definitions**

   Trainer – person who undertakes dog training.

   Instructor – person who undertakes dog training and people training.

   Handler – person deployed to work with the dog as a team.

2. **Criteria**

   The following criteria were set to ensure that all Covid-19 Detection Dog trainers, instructors and handlers are of suitable quality and experience to meet the training standards.

2.1 Trainer / Instructor criteria:

i. Trainers/instructors must be competent in the knowledge, safe handling and storage of training materials, including the use of PPE.

ii. Trainers/instructors must have a proven background in the handling and training of detection dogs.

iii. Instructors must have demonstrable competence in teaching people as well as dogs.

Instructors must have knowledge and skills in theory training relating to scent, environmental effects, learning theories, behaviour, welfare and veterinary care.

2.2 Handler criteria:

i. Handlers must be competent in the knowledge of working safe with their dog in the search environment including the use of PPE.

ii. Handlers must have a proven background in the handling of detection dogs.

iii. Handlers must have skills in theory relating to scent, environmental effects, working in a safe manner with their dogs, able to read their dog’s behaviour and ensure welfare and care.

B. Dog training:

Anecdotally dog’s olfactory sense has been known to be different from humans, in that where humans smell the whole scent picture (e.g. a hamburger), the dogs can smell each constituent`s scent in the scent picture, e.g. the meat, the bread, the sauce, tomato, lettuce

etc. in the hamburger. The training of the dogs was done in three stages. The following definitions and guidelines were set for consistency and reproducibility of the procedures.

1. Definitions

- Search stands: the apparatus in which the samples are held and where the dog searches – See Figure 1.
- Line-up / run: each set up of samples in the search stands into their given positions. A new line up is generated by the moving or replacing the samples or their positions. See Figure 2.
- Session: training session containing one or more line-ups
- Blank run: a line up where there is no positive sample.
- Training samples: all samples used in the training process= mock samples, positive and negative samples which were collected in the hospital from persons with a known Covid-19 status.
- Positive Sample: a filter that has been collected by placement under an individual armpit that is confirmed Covid-19 positive by PCR test.
- Negative Sample: a filter that has been collected by placement under an individual armpit that is confirmed Covid-19 negative by PCR test.
- Mock Sample: a naïve filter which has not been exposed to any scent or sweat in any form.
- Operational samples: a filter which has been collected by placement under an individual armpit samples taken at an operational setting where it was unknown if the person Covid-19 positive or negative. The sample was screened by the dogs earlier and PCR result came later.
- Imprinting: the process of the dog learning a target odour.
- Indication Behaviour: the behaviour a dog will perform to notify the handler it has located the target odour.
- Positive reinforcement: the process of encouraging or establishing a pattern of behaviour by offering a reward when the behaviour is strengthened.
- Positive conditioning: the learning process of introducing a dog to something new through positive reinforcement.

- Variable reward schedule: We rewarded the dog using a variety of methods (including verbal praise, tactile praise, play/toy, or no reward) using a non-constant ratio of reward to non-reward for positive response.
- PPE: Personal Protective Equipment

2. Guidelines

i. All dogs training will be supervised by the trainers and instructors.

ii. Samples for training purpose will be placed into search stands by the Trainer/Instructor or assistant knowing the content of each sample, and handlers being unaware of the format of the line-up i.e. a single blind search.

iii. Operational samples will be placed into search stands by the Trainer/Instructor or assistant without them knowing the content of each sample, and handlers also unaware of the format of the line-up i.e. a double blind search.

iv. Training must reflect operational conditions and be free of hazards. This includes the use of appropriate PPE for all personnel involved in Covid-19 Detection training or operations as detailed below

- Over suit, including head cover
- Footwear cover
- Double gloves
- Double face masks
- Face Shield

Equipment used for training must be cleaned with particular attention to any equipment that has been exposed to target substances. On exiting the screening room all staff must proceed through a sanitisation spray. All PPE was disposed of in clinical waste containers.

v. Training methods used must maintain animal welfare and safety by not allowing any physical or mental injury. Training must be stopped if the dog shows signs of injury, distress or fatigue.

vi. A marker, e.g. clicker, may be used during training, but is not obligatory.

vii. A variable reward schedule should be used to reward the dog to keep expectation high and increase working drive.

viii. Recording

All training must be recorded with the following information as a minimum:

1) Dog Details
2) Handler/Trainer Details
3) Location of training
4) Description of positive samples
5) Description of negative samples
6) Description of line-up showing what sample is in each stand
7) Results – Indication/No Indication/False Reports/Search Behaviour
8) Comments

3. In-house Training Process

The process of Covid-19 Detection Dogs training is described below:

3.1. Training search pattern on search stands

Timeframe: this training phase was completed in one day containing three sessions.

It was ensured before the training that the dogs understood basic obedience commands such as sit, wait, and loose (drop the toy), along with lead walking behaviour.

The dogs were taught the search behaviour, starting with six search stands. Since the dogs were trained on detection of explosives, we started with placing an explosive training sample into an empty stand to encourage the dog to target this specific search stand. The explosive training samples were sometimes replaced by the dog's reward toy. The dogs were taught to place their head into each stand, in a line of six search stands, sequentially, in order to condition them to perform a methodical pattern starting the search at search stand one and complete at stand six. After several sessions the dogs understood the required search pattern, so we continued to the next step.

3.2. Imprinting

A- IMPRINTING PHASE ONE

Timeframe: this training was completed in two days containing average of three sessions per day, consisting of four line- ups on average.

During imprinting phase, three stands were placed in the line-up, where one stand had a positive sample inside, and the remaining two were empty stands. The dog was rewarded with the toy when he showed an interest in the search stand containing the positive sample. The positions of positive and empty search stands were changed and the process was repeated, thereby creating an association between the toy and the positive sample smell. When the dog was showing recognition behaviour of the positive sample, the dog was commanded to sit and was rewarded. In this manner, the dog's behaviour was shaped to give a sit indication when smelling the positive sample. In each session, multiple positive samples were used. After each session, the stands were cleaned and wiped with paper towels.

Twenty positive samples were used.

B- IMPRINTING PHASE TWO

Timeframe: this training was completed in two days containing average of three sessions per day, consisting of four line-ups on average.
A total of 24 positive samples and 192 mock samples were used.

Once the dogs understood this process, we continued with three stands containing one positive sample and two mock samples, The dog was rewarded with the toy when it showed an interest or an indication in the search stand containing the positive sample. The positions of positive and mock samples were changed and the process was repeated, thereby creating an association between the toy and the positive sample smell.

C- IMPRINTING PHASE THREE

Timeframe: this training was completed in one week, five days training consisting of three training sessions per day, where each session contained an average of four line-ups.

Once the dogs understood this process, we continued with three stands containing one positive sample and two negative samples, where again the dog was rewarded for a correct indication on the positive sample. If the dog showed interest in the negative sample,

no reward was given to the dog, from where the dog learned that the negative samples had no value, and the dog continued his search.

D- IMPRINTING PHASE FOUR

Timeframe: this training was completed in four weeks, five days per week training, containing average of three sessions per day; each training session consisted of an average of four line-ups.

After a successful completion of the previous training phase the number of search stands was increased to six, i.e. one search stand contained a positive sample and five stands contained negative samples. Each session contained an average of six line-ups and in each line-up, the position of the search stands containing positive and negative samples was changed randomly. Besides changing their position, the positive and negative samples were also frequently replaced by fresh, new samples to ensure the dog would not indicate on the specific human scent.

Due to false positive detection, the search stands were cleaned with paper towel and after each session with a water steam cleaner.

After completion of the above step, the training continued with line-ups containing all negative samples, teaching the dogs to ignore these samples. If the dogs showed interest or indication, and dogs learned to continue the search. After successful completion of a true negative line-up, the dogs were randomly rewarded by a verbal praise or in specific cases rewarding the dog with their toy.

A total of 234 positive samples and 144 negatives samples were used in the imprinting phase three and four.

E- VALIDATION PROCESS

After completion of the training phases the teams were moved to a UAE hospital, where samples were prepared of twenty individuals tested Covid-19 positive and twelve individuals tested Covid-19 negative by PCR tests.

The dog teams screened the samples on the hospital location (single blind) where the results showed above 90% detection rate.

3.3 Operational Training & Testing

During this stage, the dogs were taken to the Operational Setting and training was given in this location to acclimatise the dogs to working in other environments. Samples were taken from randomly selected persons on site and stored in an airtight container in a holding area next to the testing room, and were presented to dogs within a maximum of two hours of collection (see Figure 3). During this phase, the operational samples were placed in the search stands, where the dog searched each sample. The behaviour of the dogs was registered by the following indicators:

Negative = no indication

Positive = positive indication

Hesitation = interest in the sample (change of behaviour, but no solid indication)

At least two dogs screened each line-up. After completion of the search of all stands, the glass jars were replaced with clean ones for the next line up. Sometimes dogs showed interest in the sample but did not give clear indication; such behaviour by dogs was termed as hesitation. In case of a hesitation by one of the two dogs, while the second dog marked the sample negative, a third or fourth dog was included for checking the sample. If three dogs gave negative indication and one dog gave hesitation, the sample was recorded as negative; if two or more dogs gave hesitation and remaining dogs gave negative indication, the sample was recorded as positive. This procedure was adopted since we intend to use dogs for screening purpose and did not want to miss any positive case. Bayesian analysis results also supported our screening procedure. On average, a team of four dogs were able to screen 80 samples in a four-hour shift. This includes setting up the materials, taking the samples, placing the samples, exchanging the samples, cleaning the search stands, and replacing glass jars. During the operational runs, we calibrated the dogs frequently with positive training samples, to maintain the motivation of the dogs.

Figure 1: Search stand assembly consisting of metallic sniffing cone and sample holding compartment

a

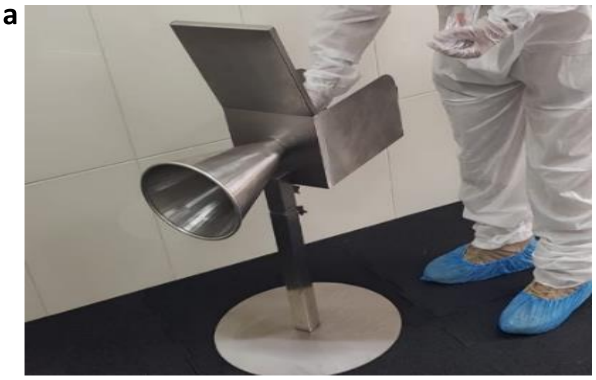

b

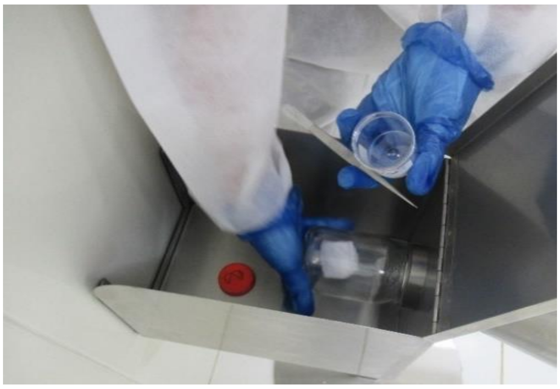

a Metal search stand showing the cone and sample holder compartment

b Placing the sample in the sample compartment behind the metal cone

Figure 2: Training room showing the search stand line-up

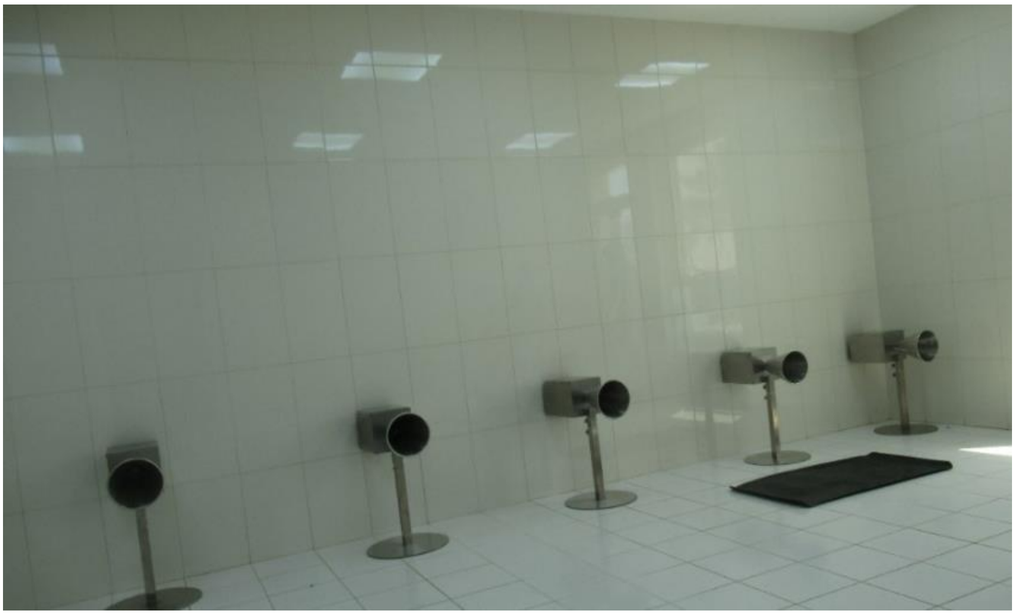

Five metal search stands are shown in the training room. Each stand has a sniffing cone and a specimen holder compartment right behind it

Figure 3: Sample collection container

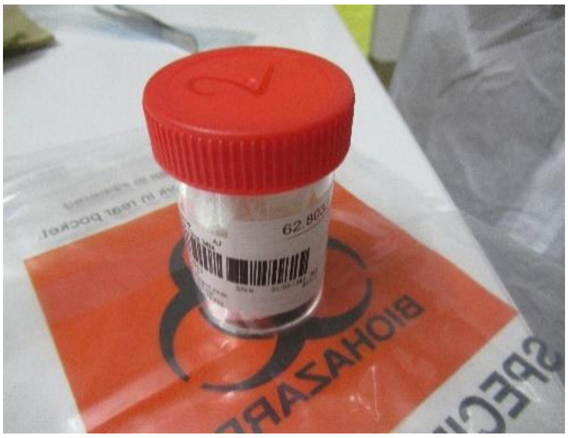

Sample collection container showing the barcode and its biohazard collection bag

Supplementary Results and Discussion Section

This part of the Supplementary information document provides the following information regarding the Bayesian analysis studies:

1. The density plots of the Bayesian model showing the sensitivity, specificity and prevalence using each of the two tests: RT-PCR and K9 dog test.
2. The convergence trace plots of the Bayesian model showing the sensitivity, specificity and prevalence using each of the two tests: RT-PCR and K9 dog test.

Diagnostic and Density Plots of the Bayesian Model

(Test 1: K9 Detection Dogs Test; Test 2: RT-PCR)

Key:

Se: Sensitivity; Sp: Specificity; [1]: Test 1: K9 Dog Detection Test; [2]: RT-PCR Test

#Density plots

Sensitivity (Se)

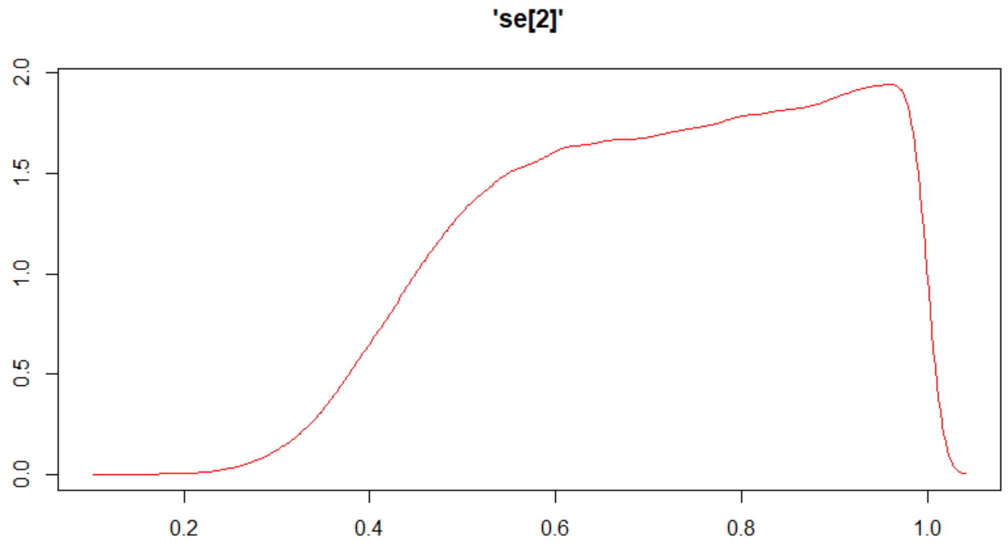

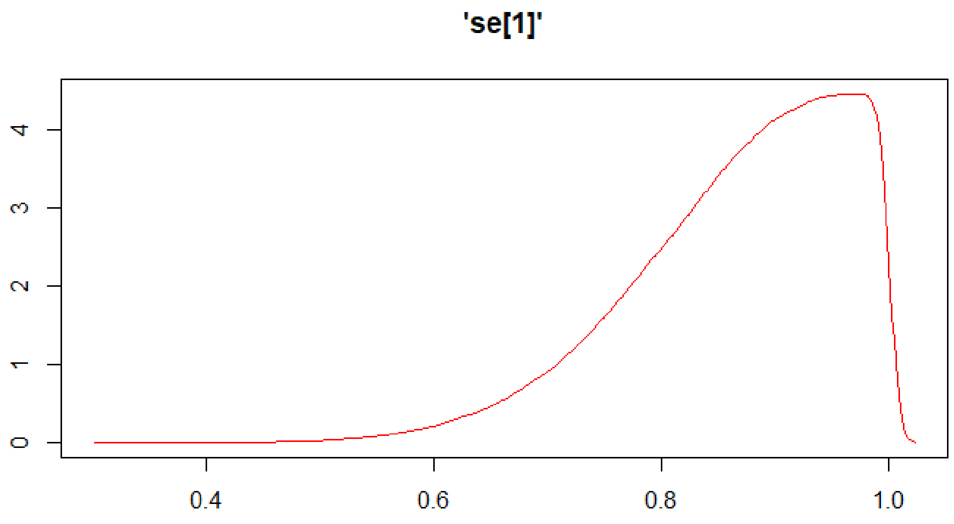

Specificity (Sp)

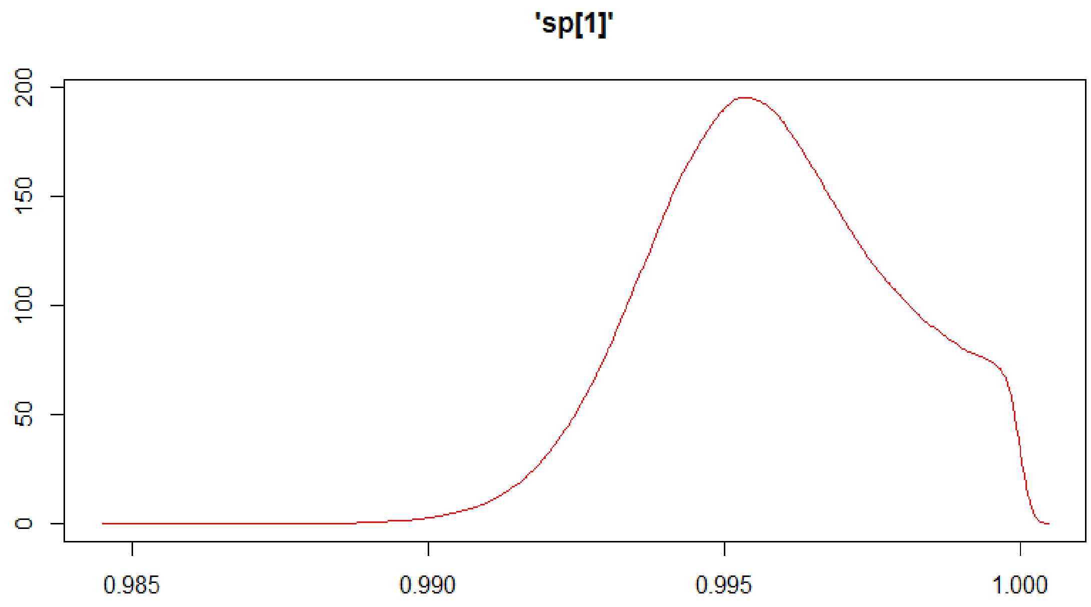
'sp[1]'
0
50
100
150
200
0.985
0.990
0.995
1.000

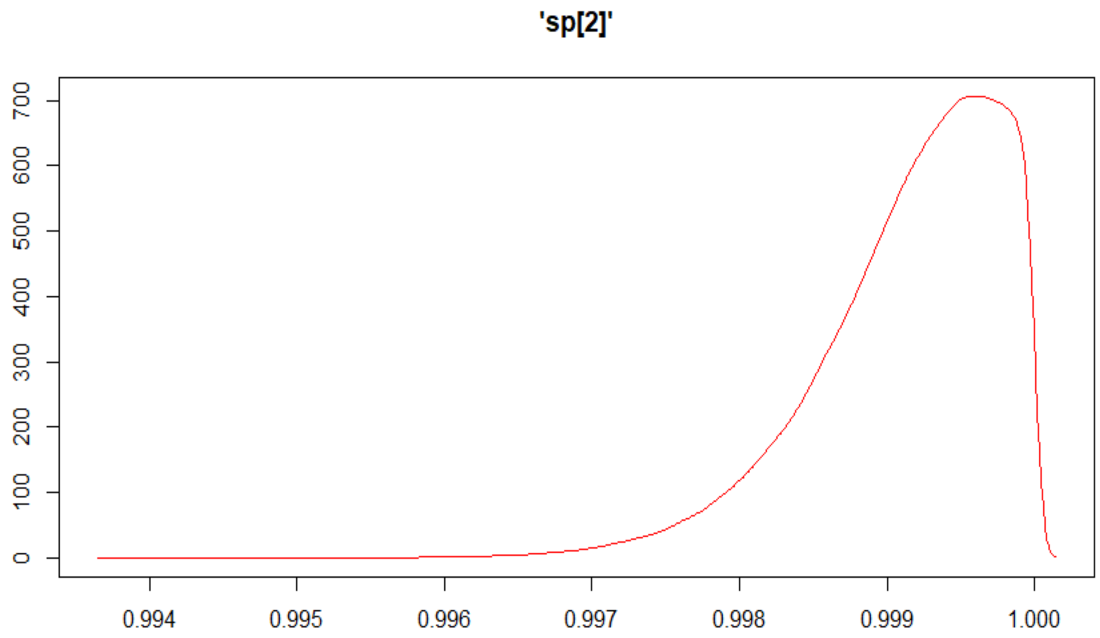
'sp[2]'
0
100
200
300
400
500
600
700
0.994
0.995
0.996
0.997
0.998
0.999
1.000

Prevalence (P)

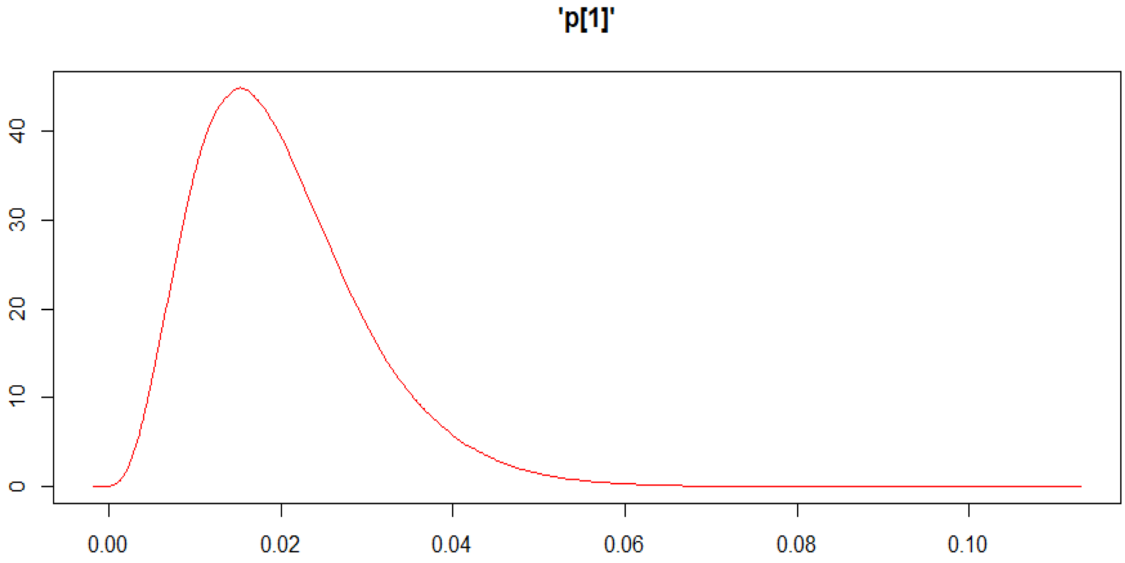

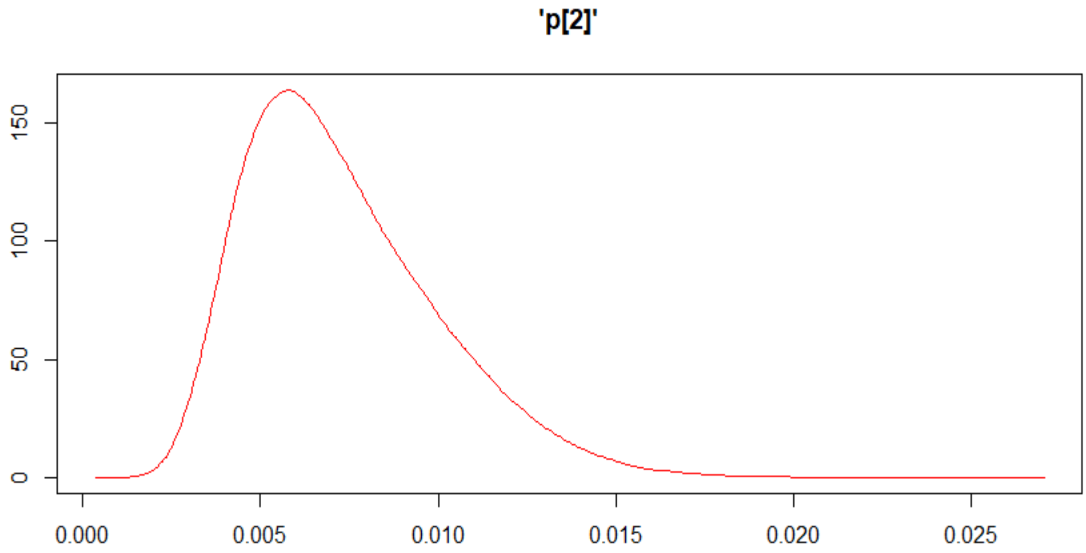

Convergence (Trace plots)

Sensitivity

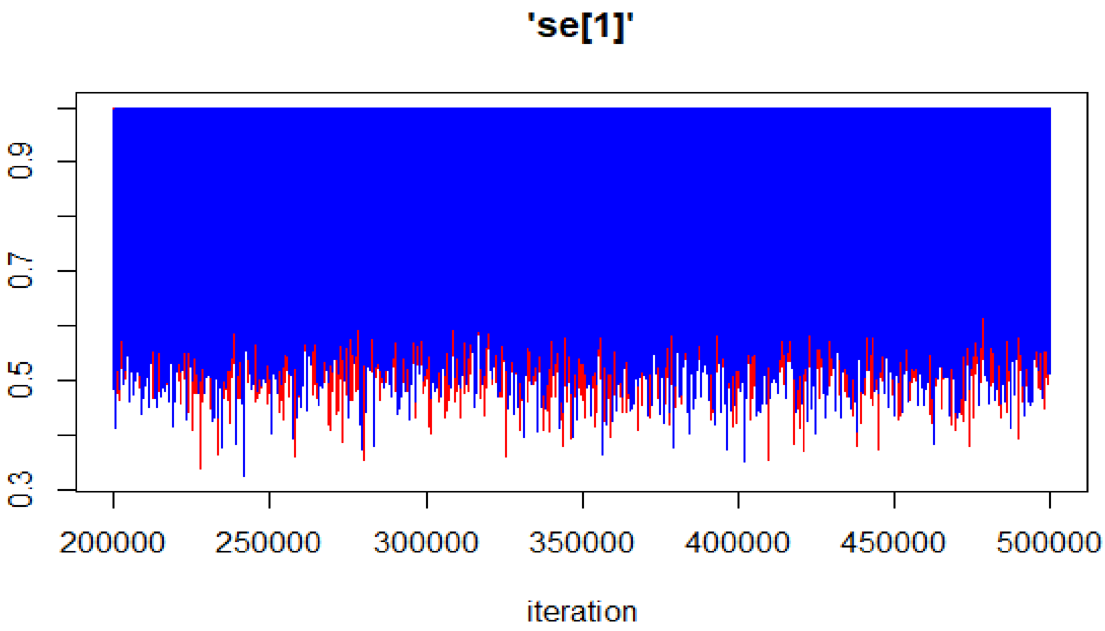

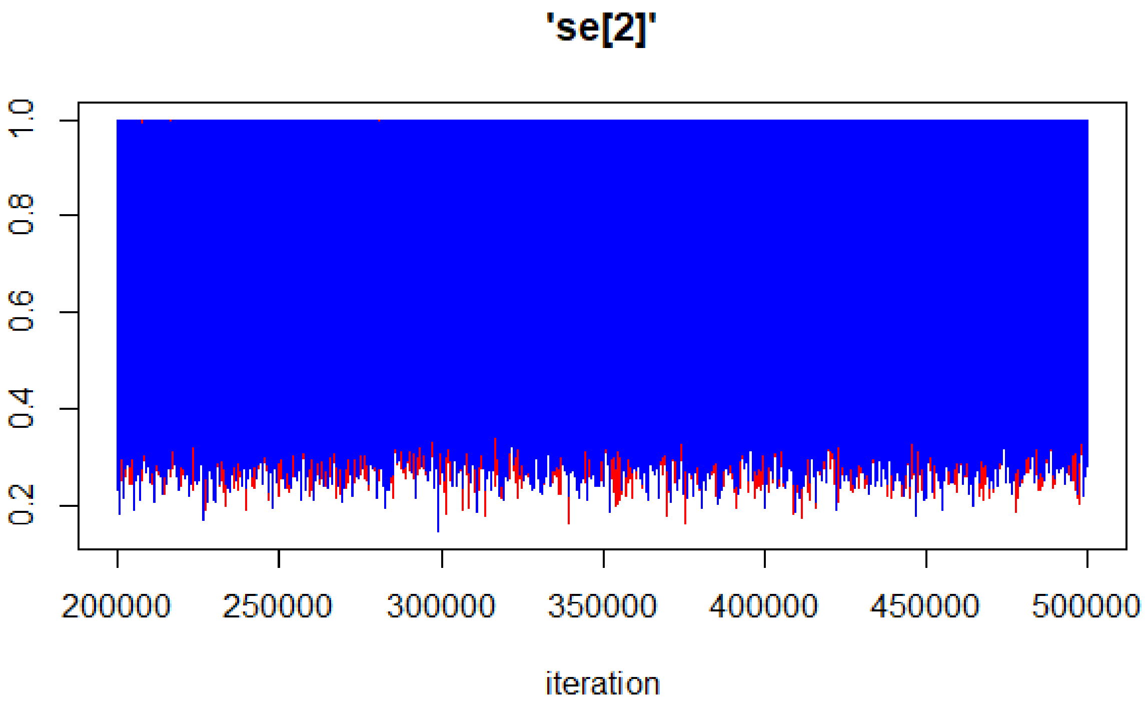

Specificity

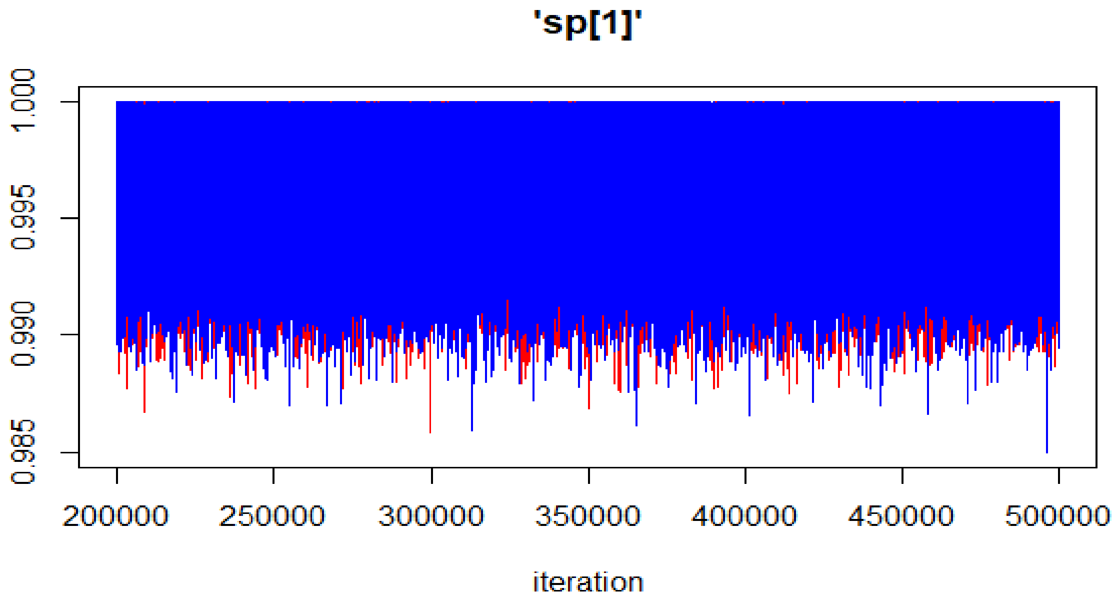

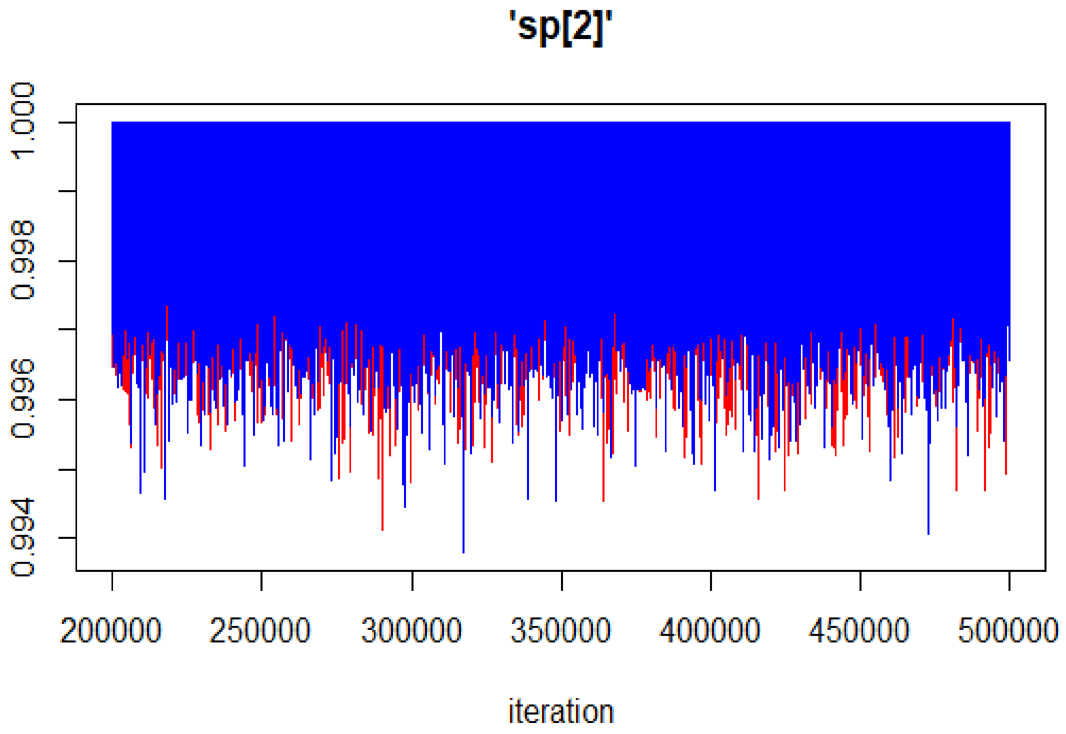

Prevalence

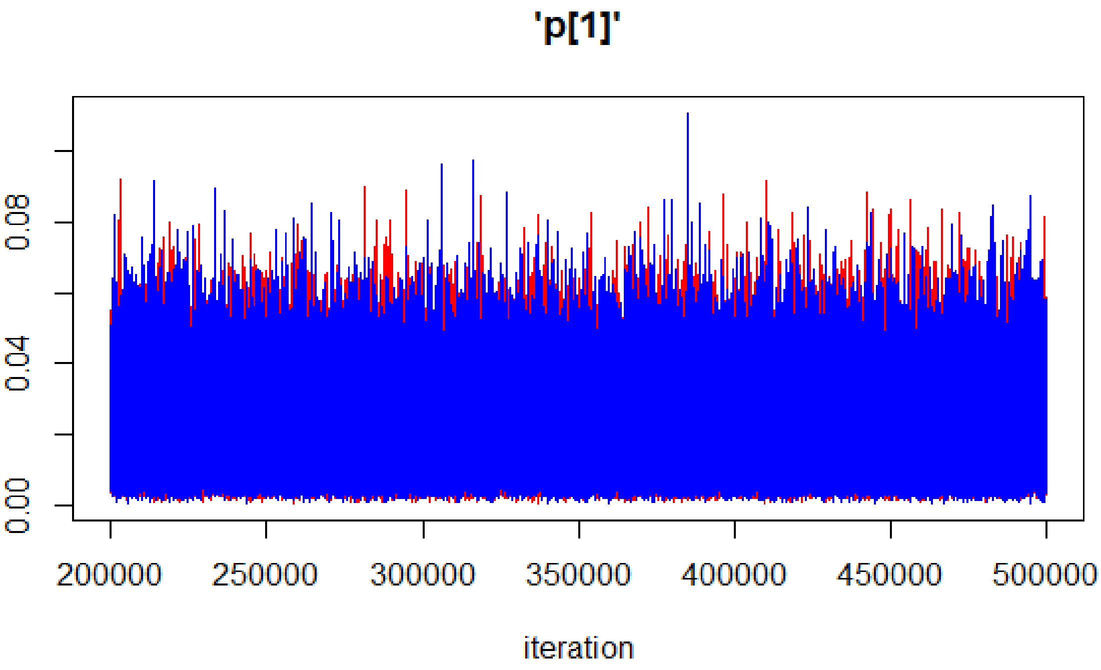

'p[1]'
0.00
0.04
0.08
200000
250000
300000
350000
400000
450000
500000
iteration

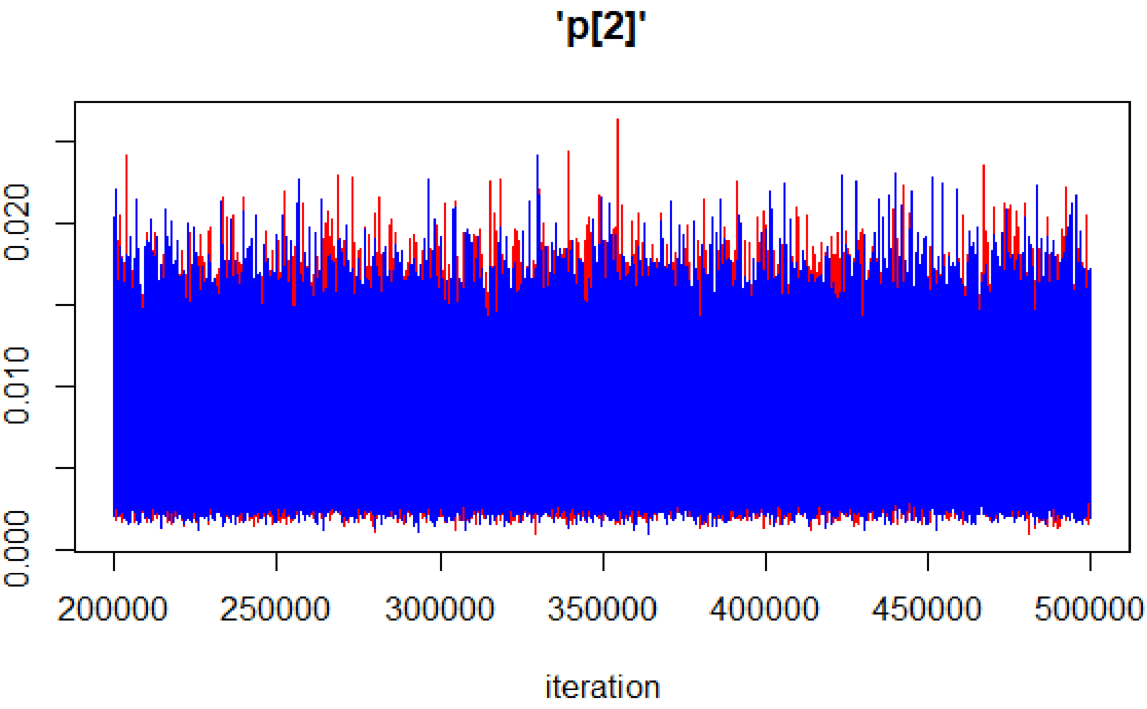

'p[2]'
0.000
0.010
0.020
200000
250000
300000
350000
400000
450000
500000
iteration

Supplementary References:

1 Centers for Disease Control and Prevention (CDC). COVID-19 and Animals. . (Updated [February 10, 2021]).

2 Jendrny, P. et al. Scent dog identification of samples from COVID-19 patients - a pilot study. BMC Infect Dis **20**, 536, doi:10.1186/s12879-020-05281-3 (2020).

3 He, X. et al. Temporal dynamics in viral shedding and transmissibility of COVID-19. Nat Med **26**, 672-675, doi:10.1038/s41591-020-0869-5 (2020).

4 Fathizadeh, H. et al. Study presence of COVID-19 (SARS-CoV-2) in the sweat of patients infected with Covid-19. Microb Pathog **149**, 104556, doi:10.1016/j.micpath.2020.104556 (2020).
