# Supplementary material for: The detection dogs test is more sensitive than real-time PCR in screening for SARS-CoV-2
Source: Commun Biol. 2021 Jun 3;4:686. doi: 10.1038/s42003-021-02232-9 (PMC8175360; doi:10.1038/s42003-021-02232-9)
Supplement: Supplementary file 3 — Description of Supplementary Files [file 42003_2021_2232_MOESM3_ESM.pdf]

Description of Additional Supplementary Files

File name: Supplementary Data 1

Description: Provides raw data for the graph presented in Figure 2 in main manuscript.

File name: Supplementary Data 2

Description: Provides the R script encoding the Bayesian latent class model, which was fitted in OpenBUGS v3.2.2 to run the Bayesian analysis.
