# Supplementary material for: The detection dogs test is more sensitive than real-time PCR in screening for SARS-CoV-2
Source: Commun Biol. 2021 Jun 3;4:686. doi: 10.1038/s42003-021-02232-9 (PMC8175360; doi:10.1038/s42003-021-02232-9)
Supplement: Supplementary file 6 — Reporting Summary [file 42003_2021_2232_MOESM6_ESM.pdf]

nature research

Corresponding author(s): Mohammed Hag-Ali

Last updated by author(s): Apr 9, 2021

Reporting Summary

Nature Research wishes to improve the reproducibility of the work that we publish. This form provides structure for consistency and transparency in reporting. For further information on Nature Research policies, see our Editorial Policies and the Editorial Policy Checklist.

Statistics

For all statistical analyses, confirm that the following items are present in the figure legend, table legend, main text, or Methods section.

n/a	Confirmed	
[ ]	[x]	The exact sample size (*n*) for each experimental group/condition, given as a discrete number and unit of measurement
[ ]	[x]	A statement on whether measurements were taken from distinct samples or whether the same sample was measured repeatedly
[x]	[ ]	The statistical test(s) used AND whether they are one- or two-sided<br>*Only common tests should be described solely by name; describe more complex techniques in the Methods section.*
[ ]	[x]	A description of all covariates tested
[x]	[ ]	A description of any assumptions or corrections, such as tests of normality and adjustment for multiple comparisons
[ ]	[x]	A full description of the statistical parameters including central tendency (e.g. means) or other basic estimates (e.g. regression coefficient) AND variation (e.g. standard deviation) or associated estimates of uncertainty (e.g. confidence intervals)
[x]	[ ]	For null hypothesis testing, the test statistic (e.g. *F*, *t*, *r*) with confidence intervals, effect sizes, degrees of freedom and *P* value noted<br>*Give P values as exact values whenever suitable.*
[ ]	[x]	For Bayesian analysis, information on the choice of priors and Markov chain Monte Carlo settings
[x]	[ ]	For hierarchical and complex designs, identification of the appropriate level for tests and full reporting of outcomes
[x]	[ ]	Estimates of effect sizes (e.g. Cohen's *d*, Pearson's *r*), indicating how they were calculated

Our web collection on statistics for biologists contains articles on many of the points above.

Software and code

Policy information about availability of computer code

Data collection	No computer codes were used for data collection; Microsoft Excel Sheets were used.
Data analysis	A Bayesian latent class model fitted in OpenBUGS v3.2.2 was used. The R Script used is included in the Supplementary Information Section

For manuscripts utilizing custom algorithms or software that are central to the research but not yet described in published literature, software must be made available to editors and reviewers. We strongly encourage code deposition in a community repository (e.g. GitHub). See the Nature Research guidelines for submitting code & software for further information.

Data

Policy information about availability of data

All manuscripts must include a data availability statement. This statement should provide the following information, where applicable:

- Accession codes, unique identifiers, or web links for publicly available datasets
- A list of figures that have associated raw data
- A description of any restrictions on data availability

Web links for publicly available datasets will be provided when it is made available. There are no figures in the manuscript that are associated with raw data. There is no restriction on data available as the accessible data has already been anonymized.

Field-specific reporting

Life sciences study design

All studies must disclose on these points even when the disclosure is negative.

Sample size	No statistical method used for sample number determination. Study duration 2 months
Data exclusions	Records not showing the geographical location were excluded
Replication	No replicates were used. One specimen was tested per participant
Randomization	No randomization was made. Individuals visiting the COVID-19 screening center for their routine mandatory testing were approached to donate a sweat sample.
Blinding	Specimens were coded before testing. No test results were available at the time of specimen collection or processing.

Reporting for specific materials, systems and methods

We require information from authors about some types of materials, experimental systems and methods used in many studies. Here, indicate whether each material, system or method listed is relevant to your study. If you are not sure if a list item applies to your research, read the appropriate section before selecting a response.

Materials & experimental systems

n/a	Involved in the study
☒	☐ Antibodies
☒	☐ Eukaryotic cell lines
☒	☐ Palaeontology and archaeology
☐	☒ Animals and other organisms
☐	☒ Human research participants
☒	☐ Clinical data
☒	☐ Dual use research of concern

Methods

n/a	Involved in the study
☒	☐ ChIP-seq
☒	☐ Flow cytometry
☒	☐ MRI-based neuroimaging

Animals and other organisms

Policy information about studies involving animals; ARRIVE guidelines recommended for reporting animal research

Laboratory animals	No lab animals were used. Working dogs were employed in the study to sniff the sweat samples.
Wild animals	No wild animals used
Field-collected samples	No samples were collected from any animals
Ethics oversight	No experiments were performed on any animals. Only working dogs were used in this study. The welfare and safety of the working dogs was ensured as per guidelines. Those are referenced in the Methods section of the manuscript.

Note that full information on the approval of the study protocol must also be provided in the manuscript.

Human research participants

Policy information about studies involving human research participants

Population characteristics	Human research participants were males, aged 18 years and above. No genotypic or disease status information was collected.
Recruitment	Individuals visiting the COVID-19 screening center for their routine mandatory testing were approached to donate a sweat sample. Agreeing participants signed a consent form before sweat collection.
Ethics oversight	As per IRB, there were no ethical issues requiring oversight. On the other hand, a paricipation consent form was signed before sweat specimen collection.

Note that full information on the approval of the study protocol must also be provided in the manuscript.
